# A global fairtrade partnership needed to address injustices in the supply chains of clean energy technology materials

**DOI:** 10.1557/s43581-024-00113-2

**Published:** 2024-09-18

**Authors:** Chinedu C. Nsude, Joshua J. Wimhurst, Ramit Debnath

**Affiliations:** 1 Department of Geography and Environmental Sustainability, University of Oklahoma, Norman, OK 73019 USA; 2 Center for Peace and Development, University of Oklahoma, Norman, OK 73019 USA; 3 South Central Climate Adaptation Science Center, University of Oklahoma, Norman, OK 73019 USA; 4 Collective Intelligence & Design Group, and climaTRACES Lab, University of Cambridge, Cambridge, CB3 0HE UK; 5 Caltech-Cambridge Climate and Social Intelligence Lab, California Institute of Technology, Pasadena, 92115 USA

**Keywords:** critical materials, energy generation, environmental impacts, lifecycle, renewable, recycling

## Abstract

**Abstract:**

Renewable sources produced close to one-third of the world’s electricity in 2023. However, a limited but growing body of research suggests rapid renewable energy development is leading to conflict and resource exploitation in energy-transitioning communities. Such injustices are attributable to the extractivist nature of renewable energy development, where raw materials, also known as Clean Energy Technology Materials (CETMs), are in limited quantities and often concentrated in resource-constrained zones in the Global South. In this perspective, we call for an urgent need for energy justice considerations in CETM’s supply chain. We used demand projection data from 2020 to 2040 to look into the effects of important CETMs like nickel, cobalt, and lithium on distributive justice. We also examined the potential of these effects to tackle systemic injustices such as conflict, labor exploitation, and transactional colonialism. Next, we analyzed global mining production data from the United States Geological Survey using a CETM life cycle lens and found that increasing demand for these materials is exacerbating restorative injustices, particularly in the Global South. Finally, building on the above evidence, we called for the creation of multi-stakeholder partnerships and the establishment of fair trade standards across the critical CETM supply chain.

**Graphical abstract:**

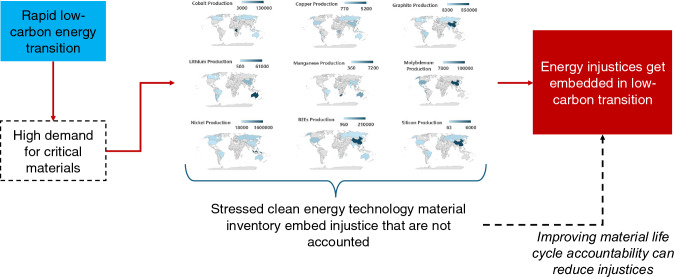

**Highlights:**

Here, we analyzed the projected demand growth for selected clean energy technology materials by 2040 relative to 2020 levels using data from the International Energy Agency, visualized their global mining production using data from the United States Geological Survey, explained how the demand for these materials is exacerbating certain injustices, and recommended multi-stakeholder partnerships across the supply chain of these materials.

**Discussion:**

- The rapid growth of renewable energy technologies is creating injustices throughout the supply chain of clean energy technology materials (CETM).
- A lack of any energy justice framework across CETMs’ extraction, processing, decommissioning, and recycling is exacerbating restorative injustices, especially in the Global South.
- By examining the projected demands and geospatial patterns for the extraction of minerals, metals, and other materials essential for clean energy technology development, the inequities faced by impoverished, marginalized, and Indigenous communities become apparent.
- We argue that if coffee can have fair trade standards across its supply chain, why can’t we have similar considerations for the CETMs?
- There is a need to include transparency in the sustainability, ethics, and energy efficiency of CETM extraction and processing through global partnerships across its supply chain.

## Need for energy justice considerations in renewable energy development

Renewable energy sources are key instruments for reducing greenhouse gas emissions by replacing fossil fuels in the energy mix. There has been a massive push globally to increase the share of renewable energy sources, and in 2023, almost one-third of the world’s electricity came from renewable sources.^1^ However, this rapid pace of renewable energy development is not always just, as evidenced by the growing incidence of conflict and resource injustices in energy-transitioning communities.^2^ Such injustices can be attributed to the extractivist nature of renewable energy development, where the raw materials, which are also known as Clean Energy Technology Materials (CETMs), are in limited quantities (like rare-earth materials) and often concentrated in resource-constrained zones in the Global South. To address such injustices with renewable energy technology, energy justice has been recognized as a critical framework for evaluating and guiding the development of CETM supply chains.^3,4^

Energy justice emphasizes the equitable distribution of both the benefits and burdens of energy production and consumption, ensuring that all stakeholders, particularly marginalized communities, have access to clean energy and are not disproportionately affected by the negative impacts associated with CETM production.^2,5^ While energy justice is critical in energy system development, evidence from the literature shows that the focus has often been on the environmental and economic aspects of supply chains, with less emphasis on social aspects. For instance, the optimization model presented by Egami^6^ emphasizes cost efficiency and environmental sustainability in supply chain design but does not explicitly address social equity aspects, such as energy affordability and accessibility.

Despite their environmental benefits, renewable energy transitions can also exacerbate social inequity in marginalized communities without enlisting an energy justice framework, such as one that considers distributional, procedural, recognition, and restorative justice dimensions.^2^ Thus, while existing literature underscores the importance of environmental sustainability and efficiency in renewable energy supply chains, there is a need for a more integrated approach that incorporates principles of energy justice. In this opinion piece, we argue that by integrating energy justice into energy transition supply chains, the benefits of renewable energy would be shared equitably, along with negative impacts being minimized across all communities involved in CETM supply chains,^7,8^ and that this integration would need a global partnership.

## Supply chain of clean energy technology materials (CETMs)

CETMs comprise various minerals, metals, and other raw materials that are essential for constructing and operating renewable energy systems. These materials are utilized in various components ranging from structural elements of wind turbines to functional materials in solar panels and batteries.^8^ The efficiency, sustainable extraction, and use of these materials are crucial for advancing clean energy technologies and the transition toward a more sustainable energy future.^9,10^ This advancement involves the use of a variety of minerals and materials. For instance, solar energy systems based on photovoltaics commonly require silicon, silver, aluminum, and copper as base materials.^11,12^ Frequently enlisted materials for wind turbines include steel (turbine tower), fiberglass/carbon fiber (blades), and rare-earth elements such as neodymium (generator magnets).^13,14^ Finally, batteries built to function as energy storage systems often use lithium, cobalt, and nickel for optimal performance and durability of electrodes.^15,16^

The supply chain of CETMs is a complex and multifaceted system involving the extraction, processing, and distribution of a wide range of raw materials.^17^ On the input side, the scaling up of clean energy technologies will significantly increase the demand for minerals.^18^ The International Energy Agency (IEA) estimates that global mineral demand for clean energy technologies could double by 2040 under a current Stated Policies Scenario (STEPS) and even quadruple under a hypothetical Sustainable Development Scenario (SDS).^19–21^ This surge in demand will place a significant strain on existing material resources and supply chains, necessitating a closer examination of the availability, sustainability, and circularity of materials in the highest demand.^22,23^ For instance, Buchholz et al.^24^ highlighted the need for research and development to cover the entire supply chain, from exploration and mining to processing, metallurgy, and recycling, thus leading to development of a circular economy.

Here we explore the supply chain and demand challenges of selected CETMs, including rare elements such as nickel, cobalt, and lithium, which could potentially face supply shortages in the coming decades. Firstly, we explored the projected demand growth for selected CETMs by 2040 relative to 2020 levels using data from the IEA’s STEPS and SDS scenarios.^21^ Secondly, we analyzed and visualized global mining production for the selected CETMs using mineral commodity data from the United States Geological Survey (USGS),^25^ discussed in detail in the following sections.

### Demand projected growth for selected CETMs

**Figure 1:**
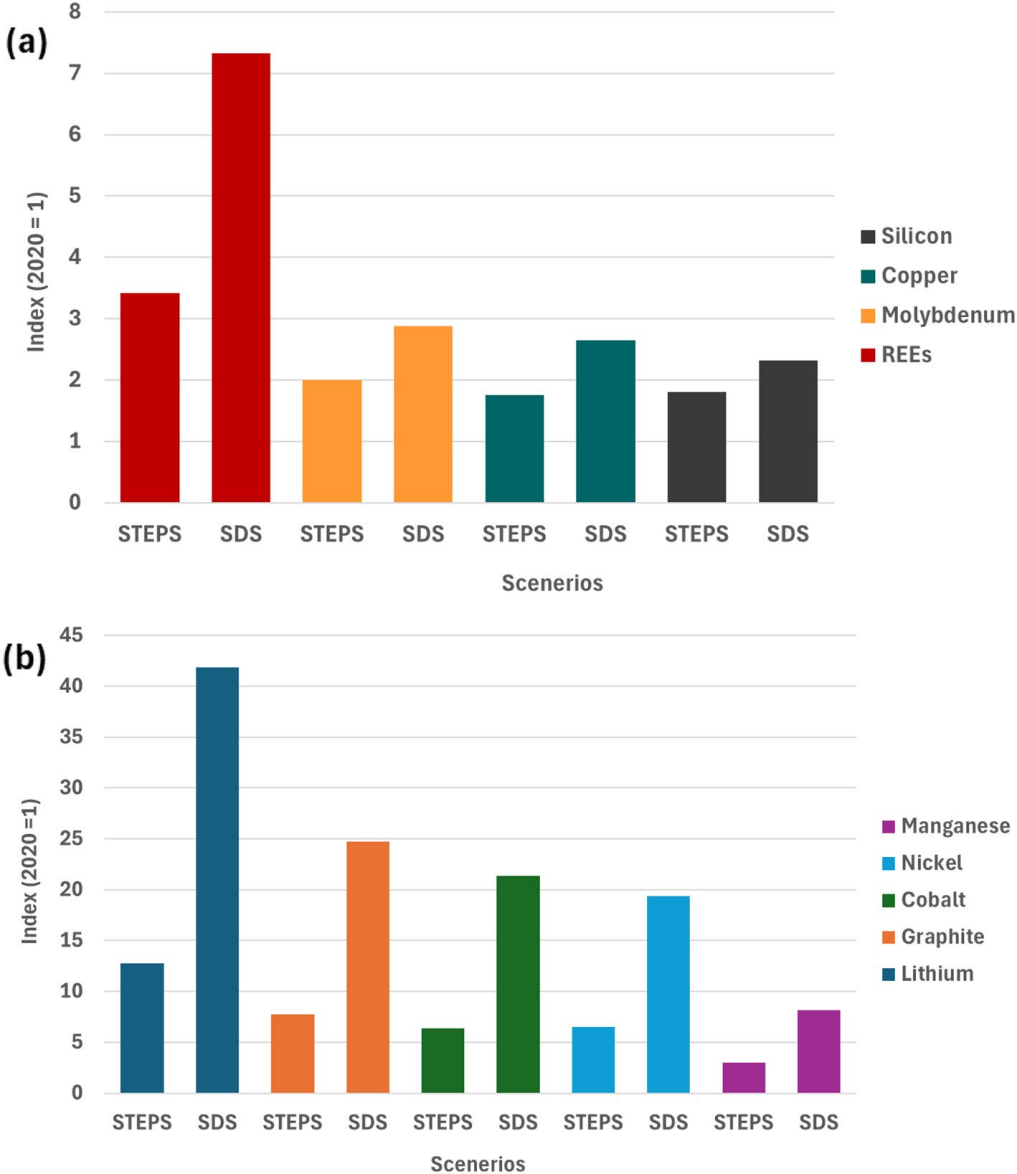
(a) Renewables and network-related minerals and (b) battery-related minerals. Projected growth in demand for selected minerals from clean energy technologies by 2040 relative to 2020 levels, based on two scenarios: *STEPS* Stated Policies scenario and *SDS* Sustainable Development Scenario. The y-axis shows the growth index, with 2020 as the base year (index = 1). Adapted from the International Energy Agency.^21^

All CETMs analyzed here have significant projected demand growth by 2040 relative to 2020 under both the STEPS and SDS scenarios.^21^ For instance, for renewables and network-related minerals, demand for Rare-Earth Elements (REEs) is projected to increase by seven times the 2020 level, molybdenum five times, copper three times, and silicon two times [Fig. 1(a)].^21^ Similarly, for battery-related minerals, lithium demand is projected to increase to approximately 42 times the 2020 level, graphite to 25 times, cobalt to 21 times, nickel to 19 times, and manganese to nine times [Fig. 1(b)].^21^ It is also noteworthy that while both the STEPS and SDS scenarios have projected demand growth for all minerals, the SDS scenario is consistently associated with much greater demand increases, reflecting more aggressive deployment targets for clean energy technologies under the latter. The substantial increase in demand for these minerals highlights the need to ensure that mining, refining, and recycling practices are sustainable and just in their support of the growth of clean energy technologies.

### Global mining production for selected CETMs

**Figure 2:**
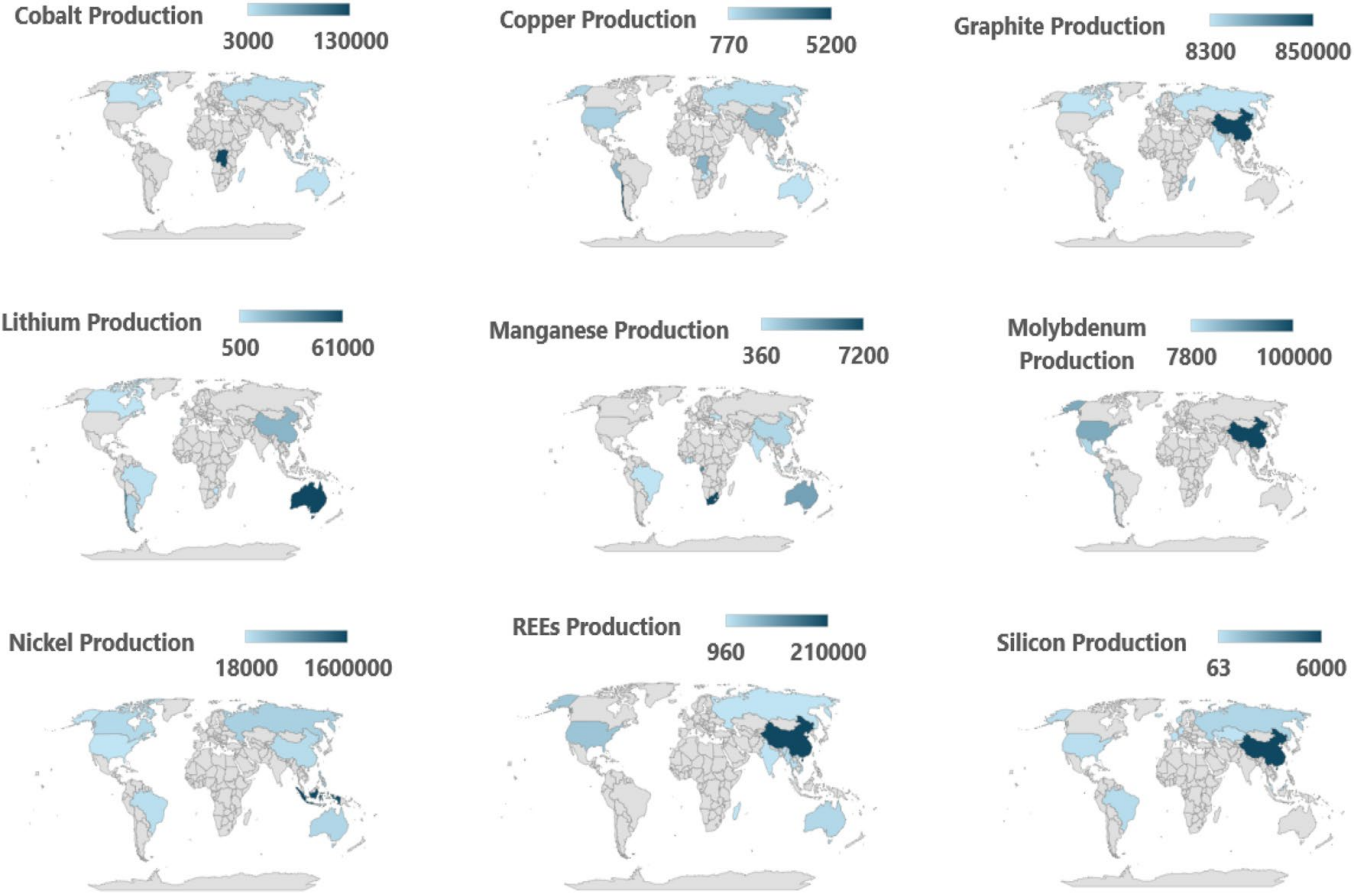
Global mining production and distribution for selected Clean Energy Technology Materials for the year 2022 (in million metric tons). Each of the maps highlights the mining production levels for a specific mineral (top-left to bottom right: cobalt, graphite, lithium, manganese, molybdenum, nickel, rare-earth elements (REE), and silicon). The color scale indicates production quantity, with lighter colors representing lower annual production. Data sourced from United States Geological Agency.^25^

The mining production of the selected CETMs was analyzed using 2022 mineral commodity data from USGS^25^ to identify patterns and trends, such as the distribution of countries that are the largest producers of specific CETMs. In addition, understanding where CETMs are produced can highlight potential vulnerabilities, dependencies, and injustices in supply chains, allowing for the development of strategies to mitigate risks. Figure 2 shows that nickel possessed the greatest production level of all selected CETMs, namely in Indonesia with its 2022 production being 1.6 million metric tons. This implied high demand for nickel could be attributed to the increasing demand for electric vehicles, as nickel is a crucial component in lithium-ion batteries. Conversely, silicon production is much lower than most other CETMs presented here, maximizing at 6,000 metric tons produced in China that same year. Although silicon is crucial for producing photovoltaic solar panels, mining production for other CETMs essential for solar panel and battery production, such as nickel, lithium, and cobalt, is much greater.

China is also a dominant producer of other CETMs, namely graphite, rare-earth elements, and molybdenum. Other leading countries in CETM production are the Democratic Republic of the Congo (Cobalt), Chile (Copper), Australia (Lithium), and South Africa (Manganese). It is worth mentioning that the authors explored the mining production of CETMs, rather than proven mineral reserves, because the former indicates the current output of mining operations based on both quantity of available materials and current supply chain dynamics that meet immediate industrial demands.

## Life cycle energy injustices of CETMs

As discussed earlier, the acceleration of renewable energy development worldwide increases the demand for CETMs, exacerbating certain injustices. For example, solar panel and wind turbine construction in its current state depends on exploitation of cheap labor and unregulated mineral extraction across the Global South.^26^ Similarly, the case of lithium extractivism on Atacameño land in Chile exemplifies this reality, with the consequent depletion of local clean water supply representing a distributive injustice to a community that receives no environmental benefit from this practice.^27^

Another example is cobalt mining in Katanga (Democratic Republic of the Congo), in which a lack of recognition justice means that women are disproportionately affected by unclean, unsafe, labor-intensive mining practices requisite to constructing batteries that enable the decarbonizing economies of affluent nations.^28^ Moreover, countries with high rates of solar energy adoption, such as Germany, have been criticized for disregarding the unjust externalities of this adoption, particularly with their reliance on cheaply extracted and processed copper from China.^29^

As for the wind energy sector, offshore wind adoption will likely result in several-fold increases in demand for necessary metals and rare-earth elements.^30^ Pressure is thus rising to explore untouched neodymium and dysprosium reserves to meet this growing demand, such as in Greenland, at the risk of procedural injustice to the Greenland Inuit, who broadly seek mining practices with “a stronger environmental and social focus.”^31^ It can ultimately be argued that the growing demand for CETMs in the extraction and processing stages of renewable energy development represents a form of “transactional colonialism,”^32^ which perpetuates systemic injustices toward less affluent nations and communities. These examples demonstrate the need to act on energy justice implications of the rapid renewable energy technologies and engage with its broad stakeholder base for appropriate policy design.

Despite renewable energy systems often being lauded as a low or zero-carbon solution for generating electricity,^33,34^ fossil fuel-based processes still dominate the manufacturing, operations, and maintenance of clean energy infrastructure. For example, the manufacturing of concrete and steel for wind turbine towers frequently depends on coal as a heat source, as does the manufacturing of solar photovoltaic panels.^35,36^ Besides providing heat, petrochemicals derived from fossil fuels are needed for lubricating a wind turbine’s gearbox,^37^ applying a hydrophobic polymer to produce self-cleaning solar panels,^38^ and constructing the electrodes of batteries intended for energy storage.^39^

Reliance on fossil fuels, whether as a CETM or otherwise, causes several forms of injustice for communities in which coal, oil, and gas extraction occurs. As one example, environmentalists in West Virginia (United States) and Laciana Valley (Spain) have faced restorative injustice due to mountain top removal for coal mining operations, resulting in a “culture of silence” that has harmed ecological protection efforts and severed cultural attachments to the land.^40^ In addition, the rise of hydraulic fracturing (“fracking”) to extract unconventional oil and gas reserves has led to multiple cases of soil and (ground) water pollution due to disposal/leakage of contaminated water used in the fracking process.^41–43^ Fracking thus poses a potential recognition injustice to communities that subsist on local soil and water supply for survival and cultural practices, such as farmers in South Africa’s Drakensburg Mountains^44^ and residents atop Pennsylvania’s (United States) Marcellus Shale.^45^ As long as the renewable energy sector depends on fossil fuels as a CETM, the maintenance and manufacturing of wind turbines, solar panels, and batteries will remain complicit in the energy injustices caused by coal, oil, and gas extraction.

Besides the manufacturing side, energy injustices relating to CETMs are also a concern following the decommission or end-of-life of renewable energy systems. Since the late-2010s, many of the planet’s oldest wind turbines and solar panels have started reaching the end of their service life, rendering them a form of hazardous waste that must either be disposed of or recycled.^46,47^ This is concerning as the expected increases in demand for CETMs in the coming decades will increase renewable energy wastes like wind turbine blades, solar panels, and battery components,^21,48^ which if not dealt appropriately can exacerbate further negative impacts and injustices to the environment and the society.

For instance, the decision to dispose of wind turbine blades in landfill sites is often motivated by the energy-intensiveness of separating and thus recycling their composite materials,^49^ though disposal carries risks of methane and volatile organic carbon release from the blades’ decaying organic components.^50^ Given how often landfill sites are located in city districts and countries characterized by cheaper, less “prestigious” land,^51^ the ecological and health consequences of turbine blade disposal would be felt most acutely by poorer, marginalized population groups (especially Black, Indigenous, and People of Color), exacerbating recognition injustice and environmental racism.^52^ Solar panels can cause similar injustices upon disposal due to the risk of toxic metals used to manufacture them leaching into local soil and water supply,^53,54^ of which incidents have occurred in China.^55^

Appropriate recycling of energy storage batteries is also important for preventing leakage of toxic materials into ecosystems,^56^ as well as being energy- and resource-intensive;^57^ hence, some injustices relating to CETMs for batteries (e.g., fossil fuel dependence, extractivism in the Global South) also do not transpire until the disposal or the recycling phase. Some of the battery-related injustices are known, while the majority of negative impacts on the environment and society are still unknown. Can the benefits of having a robust energy storage infrastructure outweigh its environmental and social costs? We do not know that yet.

The reliance of a renewable energy system on CETMs, whether minerals or metals for supporting infrastructures or fossil fuels for energy provision and maintenance, produces injustice at multiple stages of a system’s life cycle; hence, the sustainability, ethics, and energy efficiency of CETM extraction and processing must be improved through global partnerships across its supply chain.

## The way forward

As the clean energy technology portfolios of countries around the world continue to grow, so will the importance of energy justice frameworks that support responsible clean energy technology materials (CETM) extraction, processing, and decommissioning/recycling. This article seeks to draw attention to the lack of emphasis on social impacts caused by energy injustice relating to CETMs (rather than economic and environmental impacts). By examining the projected demands and geospatial patterns for the extraction of minerals, metals, and other materials essential for clean energy technology development, the inequities faced by impoverished, marginalized, and Indigenous communities become apparent.

The pursuit of more affluent nations and corporations to reduce their dependence on fossil fuels, while also profiting from a decarbonizing electricity sector, is frequently at the expense of nations and communities that natively possess the raw materials for constructing technologies marketed as low or zero-carbon alternatives. Indeed, many of the nations that contain abundant CETMs crucial for renewable energy system development (e.g., Chile, Indonesia, Democratic Republic of the Congo) are not target markets for selling these systems, instead perpetuating transactional colonialism that maintains them as primary producers at a more vulnerable socioeconomic status. It is highly likely that treatment of communities native to the largest CETM reserves will worsen (e.g., elimination of potable water, lost connection to land due to mining and disposal operations, health casualties caused by pollution exposure) should the clean energy transition continue in its current unjust state. The transition to a low or zero-carbon global energy system that embraces energy justice will thus require innovation, sustainability, and respect for human life at all stages of a clean energy technology’s life cycle.

Implementation of energy justice frameworks can enable community, nation, and worldwide progress toward energy transitions, such as through implementation of a Critical Restoration Geography (CRG) framework previously proposed by the authors.^2^ This framework posits seven strategies for the pre-emptive avoidance of documented injustices caused by low-carbon/renewable energy system development, whether of CETMs or otherwise. These strategies include place-based approaches that process and develop CETMs with recognition and acceptance of the characteristics and histories of development sites, and acknowledging the connections between social and ecological systems by considering the interdependence between biodiversity and CETM development. While not quantitative by design, the CRG framework serves as indicators for stakeholder evaluation of the social and environmental life cycle impacts of CETMs.

In quantitative terms, a social life cycle assessment (LCA) framework^58^ can enhance accountability and transparency throughout the CETM life cycle, thereby strengthening the application of energy justice principles throughout the supply chain. In this context, a social LCA approach allows for maximum justice across energy systems, from CETM extraction to low-carbon energy device and infrastructure deployment. This approach enables the identification of injustices and their variations among individual stakeholder categories, as well as the design of intervention points across the same energy system’s life cycle. Consequently, it provides a chance to assess potential energy justice issues related to CETMs, bolstering the development of infrastructure and human resources as we shift to low-carbon energy sources and debate the implementation of novel technologies at scale.

Therefore, the way forward is to establish multi-stakeholder partnerships across the supply chain of CETMs and their supporting infrastructure, have their stories heard and recorded, and enforce appropriate energy justice frameworks throughout the life cycle of renewable energy projects. We were wondering: if coffee beans, through the Fairtrade Standards, can have ethical accountability across their supply chain, why can’t renewable energy products and services?

## Data Availability

Not Applicable.
